# Trehalose Attenuates Oxidative Stress and Endoplasmic Reticulum Stress-Mediated Apoptosis in IPEC-J2 Cells Subjected to Heat Stress

**DOI:** 10.3390/ani12162093

**Published:** 2022-08-16

**Authors:** Fan Mo, Xu Zhou, Mengting Yang, Leyi Chen, Zhining Tang, Chong Wang, Yanjun Cui

**Affiliations:** Key Laboratory of Applied Technology on Green-Eco-Healthy Animal Husbandry of Zhejiang Province, China-Australian Joint Laboratory for Animal Health Big Data Analytics, Zhejiang Provincial Engineering Laboratory for Animal Health Inspection & Internet Technology, College of Animal Science and Technology & College of Veterinary Medicine of Zhejiang A&F University, Zhejiang A&F University, Lin’an, Hangzhou 311300, China

**Keywords:** heat stress, endoplasmic reticulum stress, apoptosis, trehalose, IPEC-J2 cells

## Abstract

**Simple Summary:**

As global warming continues, the increased frequency and intensity of high ambient temperatures imposes heat stress (HS) on farm animals and human beings. Pigs are susceptible to heat exposure; decreased performance and increased morbidity due to HS give rise to economic losses for the swine industry. HS induces oxidative stress and apoptosis, both of which are associated with compromised intestinal barrier integrity. Trehalose (Tre) is a natural disaccharide that is considered a health-promoting food owing to its antioxidant and anti-inflammatory effects and its molecular chaperone ability to inhibit protein denaturation. The present study aimed to determine the optimum trehalose level for alleviating HS-induced intestinal porcine epithelial cell (IPEC-J2) insults and to uncover the molecular mechanism of the protective effects of Tre. This study demonstrated that 10 mM trehalose can significantly attenuate HS-induced IPEC-J2 cell impairment and that the mechanism is related to trehalose alleviating oxidative stress and endoplasmic reticulum stress-mediated apoptosis.

**Abstract:**

This study was carried out to investigate the effects of trehalose (Tre) on antioxidant capacity, endoplasmic reticulum stress (ERS) response and apoptosis of heat-stressed intestinal porcine epithelial cells (IPEC-J2). IPEC-J2 cells were cultured at 37 °C until the end of the experiment (control, CON); exposed to heat stress for 2 h (43 °C, HS); or pretreated with 0.1, 1, 5, 10, and 15 mM trehalose at 37 °C for 4 h prior to heat stress exposure for 2 h. The optimum level of trehalose for protecting against HS-induced cell injuries was determined to be 10 mM, as evidenced by the highest cellular viability and lowest malondialdehyde (MDA) content and lactate dehydrogenase (LDH) activity. Based on these, IPEC-J2 cells were divided into three groups: the first group was cultured at 37 °C until the end of the experiment (control, CON); the second group was exposed to heat stress for 2 h (43 °C, HS); the third group was pretreated with 10 mM trehalose for 4 h at 37 °C prior to heat stress exposure for 2 h (Tre + HS). The reactive oxygen species (ROS) content, superoxide dismutase (SOD) activity, mitochondrial membrane potential (MMP) changes, and expressions of the manganese superoxide dismutase (SOD2), ERS and apoptosis-related proteins were determined. Compared to the CON group, HS significantly increased ROS generation (*p* < 0.01), decreased SOD activity (*p* < 0.05), and downregulated protein expression of SOD2 (*p* < 0.01). Compared to the HS group, Tre supplementation reduced ROS levels and increased SOD activity and SOD2 expression to the levels that were comparable to the control (*p* < 0.05). The HS-induced ERS response was evidenced by the increased protein expressions of glucose-regulated protein 78 (GRP78) (*p* < 0.01), eukaryotic translation initiation factor 2α (p-eif2α) (*p* < 0.01), transcription activator 4 (ATF4) (*p* < 0.01), and the protein expression of C/EBP homologous protein (CHOP) (*p* < 0.01), which were the four hallmarks of ERS. The Tre + HS group showed lower expressions of GRP78 (*p* < 0.01), p-eif2α (*p* < 0.01), ATF4 (*p* < 0.01), and CHOP (*p* < 0.01) than that of the HS group. Tre pretreatment attenuated HS-induced mitochondrial apoptosis in IPEC-J2 cells, demonstrated by the increased MMP and decreased proapoptotic proteins active caspase 3, Bax, and cytochrome c (Cyt c). Taken together, trehalose can protect against HS-induced oxidative damage and endoplasmic reticulum stress-mediated apoptosis in IPEC-J2 cells. These data may provide a nutritional strategy for alleviating heat stress in pig production.

## 1. Introduction

The increasing risk of high ambient temperatures, due to the worsening of climate warming, causes heat stress (HS) in human beings and livestock, affecting health and development. Specifically, pigs are faced with greater challenges under heat exposure due to their limited sweat glands for heat dissipation and high metabolic heat production [1,2]. Heat stress leads to reduced performance, poor meat quality, and increased morbidity and mortality, all of which cause economic losses for the pig industry [3]. Physiologically, the gastrointestinal tract is vulnerable to HS as a result of the deficiency of oxygen and nutrients in the blood, which are supplied to the peripheral tissue for heat dissipation [4], compromising intestinal barrier function [5,6]. Therefore, this may increase the intestinal permeability, allowing bacteria and their harmful metabolites to enter the blood, triggering a systemic inflammatory response [7].

Heat stress is known to disrupt the intracellular redox balance between the generation of reactive oxygen species (ROS) and the antioxidant system, affecting enzymes including superoxide dismutase (SOD), catalase (CAT) and glutathione peroxidase (GSH-PX) and leading to oxidative stress in livestock animals [8,9,10]. Our previous study suggested that chronic HS decreases the activities of SOD and CAT in finishing pigs [11]. In vitro, HS accounts for increased ROS generation and decreased superoxide dismutase 2 (SOD2) expression in intestinal porcine epithelial cells (IPEC-J2) [12]. Furthermore, in the jejunum of pigs and IPEC-J2 cells, HS-induced oxidative stress is a reason for epithelial cell apoptosis, which is associated with intestinal barrier dysfunction [7,12].

Both heat exposure or ROS can lead to the accumulation of misfolded proteins during synthesis and processing in the endoplasmic reticulum (ER), initiating an unfolded protein response (UPR), which is also known as an endoplasmic reticulum stress (ERS) response [13,14]. This adaptive mechanism depends on ER transmembrane sensors ire1, perk, and atf6 to reduce global messenger RNA (mRNA) translation, enhance misfolded protein degradation, and increase expression of ER chaperones such as GRP78 [15,16]. However, if ERS is too severe to recover, the UPR pathway switches from pro-survival to pro-apoptotic signaling. The UPR signal enhances the phosphorylation of eif2α and mediates the aggregation of ATF4 and its downstream pro-apoptotic transcription factor CHOP to the nucleus, and the pore-forming proteins such as Bcl2, Bax, and Bim are transmitted to the mitochondria to trigger cell apoptosis [17,18,19]. Our previous studies have suggested that HS induces ERS-mediated apoptosis by activating p-eif2α-ATF4-CHOP signaling in IPEC-J2 cells [20].

Trehalose (Tre) is a nonreducing disaccharide composed of two α,α-1,1-glycosidic bonds that undergo D-glucose dehydration and condensation, and is widely distributed in many nonmammalian organisms, including plants and microorganisms [21,22]. Numerous studies have reported that Tre has an antioxidant role in protecting against lipid peroxidation [21,23]. Mechanically, Tre could alleviate oxidative stress-induced damage to the spleen by activating the Nrf2 signaling pathway [24]. Recently, Tre was reported to act as a molecular chaperone and could prevent protein misfolding in the ER under stress [25]. Tre alleviates ERS by inhibiting GRP78-ATF6 signaling in dopaminergic SH-SY5Y cells [26]. Supplementation of Tre can alleviate hepatic ERS in aged mice by inhibiting ATF4 signaling [25]. These in vitro and in vivo studies demonstrated a critical role for Tre in mediating ERS. Based on these findings, we hypothesized that Tre might have a protective effect against HS-induced injuries in IPEC-J2 cells. Hence, we established an in vitro acute HS-induced intestinal damage model using IPEC-J2 cells that were exposed to high temperatures (43 °C) for 2 h. We evaluated the effects of Tre on redox balance, ERS response, and mitochondrial apoptosis signals in heat-stressed IPEC-J2 cells.

## 2. Materials and Methods

### 2.1. Cell Culture and Treatments

The IPEC-J2 cells were a gift from Prof. Haifeng Wang at Zhejiang University. The cells were seeded in DMEM/F12 culture medium (KeyGEN, Shanghai, China) containing 10% fetal bovine serum (Gibco, Waltham, MA, USA) and incubated in a 5% CO_2_ humidified incubator at 37 °C. The media was renewed every day. The well-grown cells in the log phase were cultured at 37 °C for 24 h in 96-well plates after resuscitation. Thereafter, IPEC-J2 cells were cultured at 37 °C until the end of the experiment (control, CON); exposed to heat stress for 2 h (43 °C, HS); or pretreated with 0.1, 1, 5, 10, and 15 mM trehalose (Sigma-Aldrich, St. Louis, MO, USA) at 37 °C for 4 h prior to heat stress exposure for 2 h. All cell samples were used to determine the cell viability, lactate dehydrogenase (LDH) release to the supernatant, and malondialdehyde (MDA) levels. All experiments were repeated 3 times.

### 2.2. Cell Viability Assay

Cell viability was determined by Cell Counting Kit-8 (CCK-8, MedChemExpress, Princeton, NJ, USA) as previously described [12]. Briefly, IPEC-J2 cells from all groups were cultured in 96-well plates at a density of 1.0 × 10^4^ cells/well with corresponding treatments. Then, 10 μL of CCK-8 solution was added to the cell culture medium, incubated at 37  °C for 45 min, and then the OD value at 450 nm was measured with a microplate reader (Thermo Fisher Scientific, Waltham, MA USA).

### 2.3. Lactate Dehydrogenase (LDH) and Malondialdehyde (MDA) Content Determination

IPEC-J2 cells from all groups were seeded in 96-well plates (1.0 × 10^4^ cells/well) with corresponding treatments. Then, the supernatant of the medium was collected for the lactate dehydrogenase (LDH, cat. no. A020) assay; the cells were used to measure malondialdehyde (MDA, cat. no. A003) levels using kits from the Nanjing Jiancheng Bioengineering Institute (Nanjing, China) in accordance with the manufacturer’s instructions.

### 2.4. Intracellular ROS Levels

Pretreatment with 10 mM Tre has the best effects in relieving HS-induced cell injuries, and thus, was selected for the following experiments. Based on this, IPEC-J2 cells were divided into three groups: the first group was cultured at 37 °C until the end of the experiment (control, CON); the second group was exposed to heat stress for 2 h (43 °C, HS); the third group was pretreated with 10 mM trehalose for 4 h at 37 °C prior to heat stress exposure for 2 h (Tre + HS). All cell samples were used to determine cellular ROS levels, SOD activity, protein expression levels, mitochondrial membrane potential, and cell apoptosis. All experiments were repeated 3 times.

Cellular ROS levels were determined according to the instructions of the ROS detection kit (Beyotime, Shanghai, China). Briefly, the DCFH-DA probe was added to the medium at a ratio of 1:1000, and then incubated at 37 °C for 1 h. The cells were washed twice with serum-free culture medium and observed with a fluorescence microscope (NTX-N3, Nikon Corp., Tokyo, Japan). Intracellular ROS levels were quantified using the mean fluorescence intensity per cell via Image J software (National Institute of Health, Bethesda, MD, USA).

### 2.5. Superoxide Dismutase Activity Assay

Superoxide dismutase (SOD) activities were detected using a commercial kit (cat. no. A001) from the Nanjing Jiancheng Bioengineering Institute (Nanjing, China) in accordance with the manufacturer’s instructions.

### 2.6. Mitochondrial Membrane Potential Assay

Mitochondrial membrane potential (MMP) was detected using the cationic dye JC-1 (Absin, Shanghai, China). IPEC-J2 cells were mixed with JC-1 dye (10 μM) for 15 min at 37 °C and then washed with PBS (pH = 7.2). Cells were imaged and analyzed with fluorescence microscopy (NTX-N3, Nikon Corp., Tokyo, Japan) to assess changes in the ratio of red to green fluorescence.

### 2.7. Flow Cytometry Detection of Apoptosis

IPEC-J2 cell apoptosis was assayed by a commercial kit (Annexin V/PI Apoptosis, BD, FACSCalibur, USA). IPEC-J2 cells were fixed in binding buffer mixed with 5 μL of PI and 5 μL of Annexin V/FITC based on the manufacturer’s protocols. After being incubated in the dark for 15 min at 25 °C, cellular apoptosis was assayed via a Becton Dickinson FACScan combined with Flowjo software (Becton and Dickinson, Franklin Lakes, NJ, USA) analysis.

### 2.8. Western Blotting

Total and cytosolic proteins were extracted from IPEC-J2 cells based on the commercial kit protocols (cat. no. KGP950 and no. KGBSP002, respectively, KeyGEN Biotechnology, Nanjing, China). Western blotting analysis was conducted as previously described by our group [27]. Then, 20 µg/well proteins were transferred onto polyvinylidene fluoride (PVDF) membranes (Millipore, Burlington, MA, USA). Thereafter, the membranes were incubated at 4 °C for 24 h with antibodies against ATF4 (cat. no. 11815), p-eif2α (cat. no. 3398), SOD2 (cat. no. 13141), active caspase 3 (cat. no. 14220), GRP78 (cat. no. 3177), CHOP (cat. no. 2895) (1:1000, CST, Danvers, MA, USA), Cyt c (cat. no. ET1610-16), Bcl2 (cat. no. EM1701-82), Bax (cat. no. ET1603-34), and β-actin (cat. no. R1207-1) (1:1000, Hua’an Biotechnology, Hangzhou, China). The membranes were infiltrated in TBST mixed with a goat anti-mouse (cat. no. HA1006) or rabbit (cat. no. HA1119) IgG-HRP-conjugated secondary antibody (1:5000 Hua’an Biotechnology, Hangzhou, China). Finally, the protein signals were specifically visualized with band density and images were quantified by densitometry using image analysis software (National Institute of Health, Bethesda, MD, USA). Relative expressions of the other proteins were normalized to the β-actin.

### 2.9. Statistical Analysis

Three independent experiments were carried out and data were expressed as mean ± standard deviation (SD). Data were analyzed using SAS statistical software (SAS Institute, Cary, NC, USA). Statistical differences between mean values were determined using a one-way analysis of variance (ANOVA) with Duncan’s multiple range test. Means for variables that do not share a common letter differ significantly (*p* < 0.05).

## 3. Results

### 3.1. Effects of Trehalose on Viability of Heat-Stressed IPEC-J2 Cells

**Figure 1:**
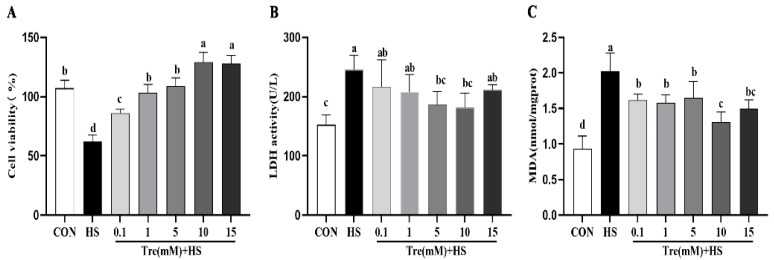
Effects of trehalose on cell viability (**A**), LDH activity (**B**), and MDA content (**C**) in heat-stressed IPEC-J2 cells. IPEC-J2 cells were cultured at 37 °C until the end of the experiment (control, CON); exposed to heat stress for 2 h (43 °C, HS); or pretreated with different concentration of trehalose (0.1, 1, 5, 10, and 15 mM) at 37 °C for 4 h prior to heat stress exposure for 2 h. Data are shown as mean ± SD (n = 3). Means for variables that do not share a common letter differ significantly (*p* < 0.05).

As shown in Figure 1A, compared to the CON group, the cell viability was significantly decreased (*p* < 0.01) in the HS group. Tre supplementation (0.1, 1, 5, and 10 mM) increased the cell viabilities in a dose-dependent manner (*p* < 0.01) compared to the HS group. Conversely, LDH release exhibited much higher levels in the HS than in the CON group (*p* < 0.05) (Figure 1B). Compared to the HS group, LDH levels were decreased in a dose-dependent manner in the Tre pretreatment groups (*p* < 0.05) (Figure 1B). Specifically, the addition of 5 and 10 mM Tre decreased the LDH release to a level that was comparable to the control (*p* > 0.05) (Figure 1B). In parallel, MDA levels were significantly decreased in the HS group as compared to the CON group. Tre pretreatment (0.1, 1, 5, and 10 mM) resulted in a dose-dependent decrease in MDA levels (*p* < 0.01) (Figure 1C) compared to the HS group. Based on these results, the 10 mM Tre pretreatment was found to have the best effects in alleviating HS-induced cell injuries, and thus, was selected for further experiments.

### 3.2. Effects of Trehalose on ROS Content and Antioxidant Capacity in Heat-Stressed IPEC-J2 Cells

**Figure 2:**
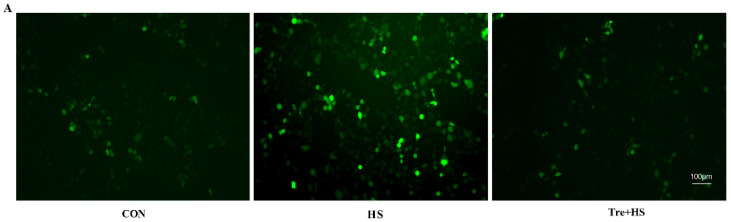

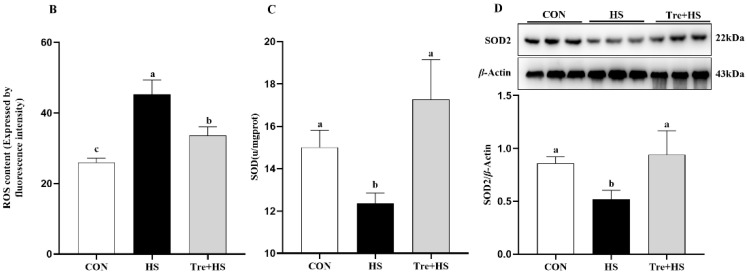
Effects of trehalose on cellular ROS level, SOD activity, SOD2 expression level in heat-stressed IPEC-J2 cells. IPEC-J2 cells were cultured at 37 °C until the end of the experiment (control, CON); exposed to heat stress for 2 h (43 °C, HS); or pretreated with 10 mM trehalose at 37 °C for 4 h prior to heat stress exposure for 2 h (Tre + HS). Cellular ROS accumulation was measured by the DCFH-DA fluorescence analysis (**A**,**B**). SOD2 activity (**C**). Expression level of SOD2 tested by Western blot (**D**). SOD2: superoxide dismutase 2. Data are shown as mean ± SD (n = 3). Means for variables that do not share a common letter differ significantly (*p* < 0.05).

ROS elimination in IPEC-J2 cells is dependent on the antioxidant defense system. Compared to the CON group, the HS group showed a significant increase in ROS content (*p* < 0.01) (Figure 2A,B). Tre supplementation contributed to the decrease in ROS levels as compared to the HS group (*p* < 0.01) (Figure 2A,B). On the contrary, SOD activity was significantly increased under heat exposure (*p* < 0.01), whereas the Tre pretreatment increased SOD activity to a level that was comparable to the control (*p* > 0.05) (Figure 2C). Moreover, Western blotting analysis demonstrated that the Tre treatment attenuated HS-induced decreases in the protein expression of SOD2 (*p* < 0.05) (Figure 2D).

### 3.3. Effects of Trehalose on Endoplasmic Reticulum Stress Response (ERS) in IPEC-J2 Cells Induced by Heat Stress

**Figure 3:**
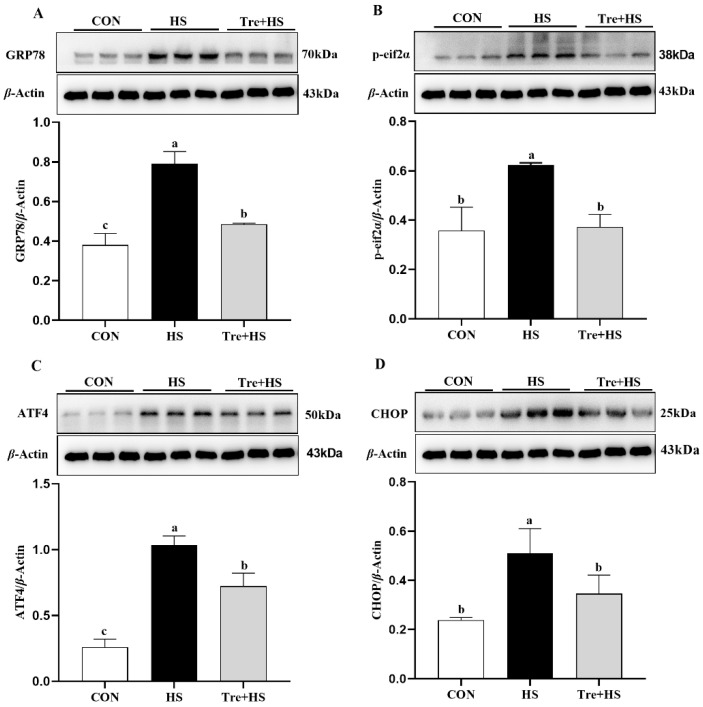
Effects of trehalose on endoplasmic reticulum stress (ERS) response in heat-stressed IPEC-J2 cells. IPEC-J2 cells were cultured at 37 °C until the end of the experiment (control, CON); exposed to heat stress for 2 h (43 °C, HS); or pretreated with 10 mM trehalose at 37 °C for 4 h prior to heat stress exposure for 2 h (Tre + HS). Western blotting analysis of the expression of UPR proteins GRP78 (**A**), p-eif2α (**B**), ATF4 (**C**), and CHOP (**D**). GRP78: glucose-regulated protein 78; p-eif2α: phosphorylated eukaryotic translation initiation factor 2α; ATF4: transcription factor 4; CHOP: C/EBP homologous protein; Data are shown as mean ± SD (n = 3). Means for variables that do not share a common letter differ significantly (*p* < 0.05).

HS-induced endoplasmic reticulum stress (ERS) response was demonstrated by the upregulation of GRP78, p-eif2α, ATF4, and CHOP, which are four hallmarks of ER stress (Figure 3). Tre supplementation decreased their expressions compared to the HS group (*p* < 0.01) (Figure 3). These findings indicate that Tre could attenuate HS-induced ERS, which is associated with the p-eif2α-CHOP signaling pathway.

### 3.4. Effects of Trehalose on Mitochondrial Membrane Potential in Heat-Stressed IPEC-J2 Cells

**Figure 4:**
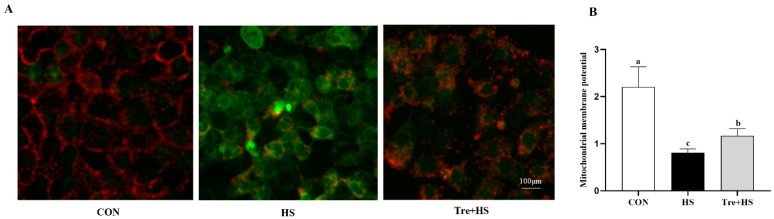
Effects of trehalose on mitochondrial membrane potential in heat-stressed IPEC-J2 cells. IPEC-J2 cells were cultured at 37 °C until the end of the experiment (control, CON); exposed to heat stress for 2 h (43 °C, HS); or pretreated with 10 mM trehalose at 37 °C for 4 h prior to heat stress exposure for 2 h (Tre + HS). Mitochondrial membrane potential was analyzed using JC-1 assay (**A**,**B**). Scale bar = 100 µm. Magnification 200×. Data are shown as mean ± SD (n = 3). Means for variables that do not share a common letter differ significantly (*p* < 0.05).

The change in mitochondrial membrane potential (MMP) can be reflected by the ratio of green to red fluorescence intensity. As shown in Figure 4, compared to the CON group, MMP was significantly decreased in the HS group (*p* < 0.01). However, the Tre + HS group showed a higher level of MMP than that of the HS group (*p* < 0.01).

### 3.5. Effects of Trehalose on Apoptosis in Heat-Stressed IPEC-J2 Cells

**Figure 5:**
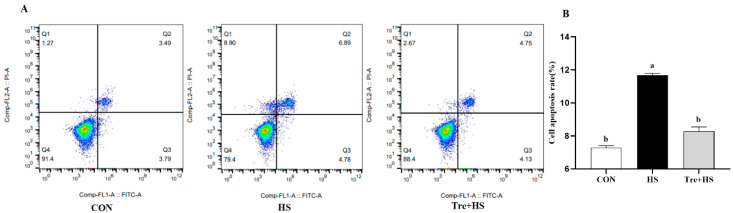
Effects of trehalose on apoptosis in heat-stressed IPEC-J2 cells. IPEC-J2 cells were cultured at 37 °C until the end of the experiment (control, CON); exposed to heat stress for 2 h (43 °C, HS); or pretreated with 10 mM trehalose at 37 °C for 4 h prior to heat stress exposure for 2 h (Tre + HS). Cell apoptosis was measured by annexin V-fluorescein isothiocyanate/propidium iodide staining and flow cytometry assay (**A**,**B**). Data are shown as mean ± SD (n = 3). Means for variables that do not share a common letter differ significantly (*p* < 0.05).

**Figure 6:**
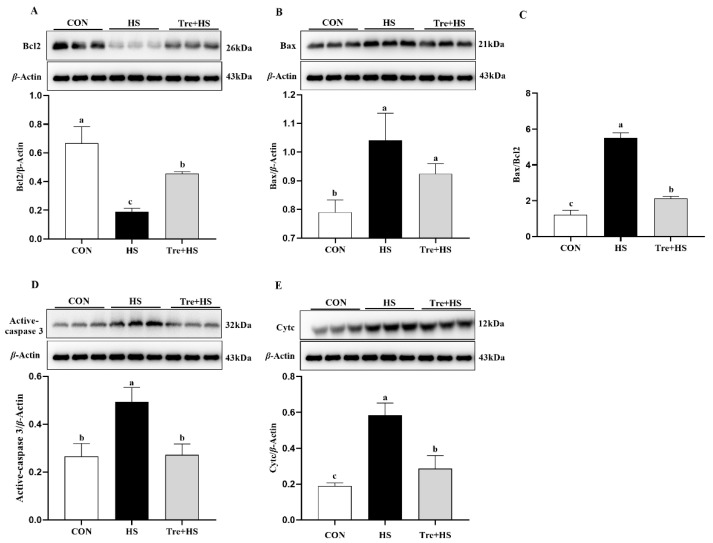
Effects of trehalose on protein expressions of apoptosis-related proteins in heat-stressed IPEC-J2 cells. IPEC-J2 cells were cultured at 37 °C until the end of the experiment (control, CON); exposed to heat stress for 2 h (43 °C, HS); or pretreated with 10 mM trehalose at 37 °C for 4 h prior to heat stress exposure for 2 h (Tre + HS). Western blotting analysis of the expression of Bcl2 (**A**) Bax (**B**), Bax/Bcl2 ratio (**C**), active caspase 3, (**D**) and Cyt c (**E**). Bax: BCL2-associated X protein; Bcl2: B-cell CLL/lymphoma 2; active caspase 3: Active cysteine aspartate-specific protease 3; Cyt c: cytochrome c. Data are shown as mean ± SD (n = 3). Means for variables that do not share a common letter differ significantly (*p* < 0.05).

Intestine epithelium is sensitive to ERS, and irreversible ER damage triggers apoptotic programs [28]. Indeed, compared to the CON group, HS induced cellular apoptosis significantly, but it was reversed by the Tre pretreatment (Figure 5). Considering that ERS-mediated cell death is dependent on transferring stress signaling from the ER to mitochondria, we evaluated expressions of pro-apoptotic proteins including Bax, Cyt c, and active caspase 3, and of the anti-apoptotic protein Bcl2. The results showed that Tre attenuates the HS-induced protein expression levels of Bax, Cyt c, the Bax/Bcl2 ratio, and active caspase 3 (*p* < 0.01) (Figure 6). These data suggest that HS-induced mitochondrial caspase activation might be a result of ER stress.

## 4. Discussion

Heat stress (HS) is known to induce oxidative stress in animals. Heat stress damages the antioxidant system, resulting in an imbalance between the production and scavenging of ROS, and in the accumulation of excessive ROS in cells [10,29]. ROS inhibits cell proliferation, increasing membrane lipid peroxidation and apoptosis [30]. Our previous studies showed that long-term HS leads to a decrease in the expression of peroxidase in the jejunum of finishing pigs and intestinal barrier dysfunction [7]. In vitro HS resulted in ROS accumulation, increased lipid peroxidation, and the decreased expression of antioxidant enzymes (SOD and CAT) in IPEC-J2 cells [20]. The HS-induced reduction in SOD and GSH-PX activities is caused by heat inactivation and oxidation of various antioxidant enzymes [31].Consistent with these results, HS reduced cell viability and increased membrane lipid peroxidation; however, 10 mM Tre significantly improved cell viability and inhibited cell damage. ROS mainly derives from aerobic metabolism in the mitochondrial respiratory chain, and MnSOD (SOD2) is the key enzyme for scavenging ROS in mitochondria [32,33,34,35]. This study found that Tre significantly increased SOD2 expression and significantly decreased intracellular ROS content. A previous study suggested that Tre could increase the expression of SOD2 and alleviate oxidative damage by activating the Nrf2/KEAP1 signaling pathway [24]. The results of this study suggest that Tre can inhibit the production of ROS and activate the intracellular antioxidant mechanism.

ROS can lead to proteins misfolding in the ER and induce an ERS response/UPR; severe or persistent ERS activates the ERS-mediated apoptosis [36,37]. In the current study, our results showed the upregulation of GRP 78, p-eif2α, ATF4, and CHOP due to heat exposure in IPEC-J2 cells, suggesting a specific activating p-eif2α-CHOP signal pathway which is a proapoptotic branch of UPR [36]. Our previous study showed that long-term HS increased GRP78 expression as a marker of ERS and intestinal permeability in the intestinal mucosa of finishing pigs [7]. In vitro, the inhibition of ER stress by 4-phenylbutyric acid, a specific inhibitor of ERS, can alleviate HS-induced IPEC-J2 cell injuries [20]. Tre administration downregulates their expressions to the levels of the control, indicating that Tre could effectively attenuate HS-induced ERS, which might be associated with the inhibition of the eif2α-CHOP signal. In line with this, Tre supplementation in drinking water can alleviate hepatic ERS in aging mice by inhibiting the phosphorylation of eif2α [25]. Tang et al. reported that Tre could alleviate the ERS-mediated apoptosis of mouse chondrocytes by inhibiting the expression of CHOP [38]. In vitro and in vivo studies have shown that Tre plays a role in attenuating ERS response and exerts a protective effect against cell injury of IPEC-J2 cells induced by HS.

Unresolved ERS as a proapoptotic signal is transited to the induction of mitochondria apoptotic cascades [19]. Consistent with a previous study in primary chick embryonic myocardial cells [39], our results showed that HS led to a significant decrease in mitochondrial membrane potential (MMP) in IPEC-J2 cells, suggesting HS-induced mitochondrial depolarization, which is a critical step in triggering apoptosis. However, Tre ameliorates the HS-induced decrease in MMP, indicating a role for Tre in the inhibition of mitochondrial apoptosis. In agreement with this, a previous study showed that Tre could ameliorate mitochondrial membrane potential collapse [38]. Indeed, the flow cytometry assay suggested that Tre attenuates HS-induced cellular apoptosis. Moreover, the pretreatment with Tre reverses the HS-induced upregulation of pro-apoptotic proteins including caspase-3, Cyt c, and Bax. Bax directly opens the mitochondrial permeability transition pore, thereby facilitating the release of Cyt c into the cytosol and activating apoptotic signaling pathways [40]. Our previous study demonstrated that the suppression of ERS, especially the blockage of the eif2α/CHOP signaling pathway, can attenuate HS-induced injuries by Bax-mediated apoptosis [19]. Therefore, the mechanism involved in the protective effects of Tre might also be what modulates ERS-mediated apoptosis. Tan et al. showed that Tre alleviates apoptosis by protecting the autophagy-lysosomal system in alveolar macrophages during human silicosis [41]. In another study, Tre could inhibit ERS-induced apoptosis by activating autophagy in rat valves [42]. In addition, Tre had a protective effect on a cadmium-induced brain injury model in rats, which is mainly related to the regulation of NRF2-mediated oxidative stress, autophagy, and apoptosis [43]. To summarize, Tre can protect against HS-induced IPEC-J2 cell apoptosis, and the mechanism is related to Tre alleviating the ERS response.

## 5. Conclusions

The present study shows that supplementation with 10 mM trehalose can significantly relieve oxidative stress and ERS-induced apoptosis in heat-stressed IPEC-J2 cells. The mechanism is related to Tre attenuating apoptosis by inhibiting the pro-apoptotic arm of UPR via the p-eif2α-CHOP signal pathway. Tre modulates the HS-induced oxidative stress response by enhancing SOD2 activities and eliminating excessive ROS. These in vitro results provide additional insights into the mechanisms of action of Tre. Future in vivo studies are warranted to confirm the beneficial effects of dietary supplementation with Tre as a strategy for alleviating HS-related intestine injury.

## Data Availability

All data presented in this study are available on request from the corresponding authors.
